# Injustice unmasked: a systematic review of social justice struggles during the COVID pandemic in Canada

**DOI:** 10.3389/fpubh.2026.1856009

**Published:** 2026-07-23

**Authors:** Charles Mensah, Samuel Bodunrin

**Affiliations:** 1 School of Social Work, Algoma University, Brampton, ON, Canada; 2 School of Business and Economics, Algoma University, Brampton, ON, Canada

**Keywords:** COVID-19, intersectionality, PRISMA, social justice, systematic review

## Abstract

**Background:**

The COVID-19 pandemic amplified social injustices through institutional system disruption among marginalized communities in Canada. This study fills an important literature gap by providing the first holistic systematic review with a theoretically grounded framework to anticipate and mitigate social injustices in future public health crises.

**Method:**

Using PRISMA 2020 framework and the databases of Web of Science, Scopus, and PubMed (*n* = 58, 2020–2023), the study analyzed the pandemic-induced inequities through the PEO framework.

**Results:**

Discrimination was identified as the most pervasive social injustice during the pandemic. Immigrants, Refugees, and Women were identified as the most vulnerable group impacted with various forms of social injustices due to the intersection between different social identities.

**Discussion:**

The novel PISIIM framework was developed to equip policy makers, social workers, and other stakeholders in anticipating and mitigating injustices in future public health crises.

**Systematic review registration:**

https://www.prisma-statement.org/prisma-2020-flow-diagram.

## 1 Introduction

The COVID-19 pandemic disrupted institutional systems and exposed deep-seated social inequalities across Canadian societies. While public health measures such as masking and physical distancing became a dominant feature of Canada’s pandemic response, the crisis also “unmasked” many systemic injustices that disproportionately affected marginalized groups.

This study critically examines how the pandemic amplified preexisting structural vulnerabilities and translated them into distinct social justice struggles faced by marginalized groups. The Pandemic-Induced Social Injustice Model (PISIIM) proposed by this study addresses a critical gap in the literature on pandemic-induced inequities by equipping policy makers, social workers, and other stakeholders with a framework to anticipate and mitigate social injustices in future public health crises.

Data from Statistics Canada show that social injustices in the Canadian society experienced a significant increase during the pandemic (63). About 62% of people from South Asian and Chinese backgrounds experienced discrimination at the onset of the COVID pandemic in Canada. The story was not different in the Black community as about 40% experienced workplace discrimination, when applying for a job or promotion (63).

While the term ‘marginalization’ is often applied when describing the exclusion of disadvantaged groups, it is essential to move beyond a descriptive label and conceptualize marginalization as a dynamic process of systemic disenfranchisement (1). A deeper look at marginalization reveals that it is a product of structural vulnerability where an individual or group’s social location is shaped by intersecting systems of power, and this systematically undermines their access to resources, fundamental rights, and opportunities (2). From the Canadian perspective, these structural vulnerabilities are embedded with historical and ongoing processes of colonization, immigration policies, and labor market stratification, which create visible patterns of inequality (3–5). Instead of viewing marginalized groups as inherently deficient or at risk, this paper presents a critical lens that posits their heightened exposure to harm is a predictable outcome of systems designed to distribute resources and risks unequally (6). The COVID-19 pandemic did not simply expose the structural vulnerabilities that are pre-existing in Canada, but exacerbated them, thereby laying bare the profound and systemic nature of social injustices in the country.

In the last few years there has been extensive research on some aspects of injustices and how it affected some groups of marginalized individuals. Also, several theories, frameworks and models have been applied to establish an understanding of the injustices that increased during the pandemic. Lately, the concept of intersectionality has been used in establishing the interconnectedness of social identities and how it increases their vulnerability to injustices. Many studies in the last few years to comprehend these phenomena have applied quantitative, qualitative, or a combination of both.

Extensive research has examined various dimensions of social injustices in Canada during the pandemic; including health disparities (7), employment impacts (8), and mental health consequences (9). While others also focused on the impacts of the pandemic on specific groups (10), no study has provided a holistic synthesis of the full spectrum of social justice struggles experienced during the pandemic, nor have they integrated multiple theoretical frameworks that explain how structural vulnerabilities are amplified through institutional disruptions.

This paper fills the gap and contributes to literature by developing a conceptual framework that explains social injustice using a pandemic-induced intersectionality framework. This study adopts an intersectional and critical lens to examine how the deep-seated structural vulnerabilities translated into specific social justice struggles experienced by marginalized communities in Canada.

## 2 Methodology

This study applies a well-documented Systematic Literature Review (SLR) to answer the research question posed in this paper. This is achieved by summarizing research publications (both qualitative and quantitative) that discuss social injustices experienced in Canada from 2020 to 2023. Preferred Reporting Items for Systematic Reviews and Meta-Analyses (PRISMA 2020) statement as suggested by Page et al. (11) is used to improve the reporting of the review paper and used to explain identification, screening and the eligibility criteria used in this study.

### 2.1 Identification

This is the initial step under the PRISMA 2020 framework, and it comprises creating the right research question, defining keywords, publication search technique, and records extracted. To develop an appropriate research question, the study applied the Population Exposure and Outcome (PEO) framework as recommended by Bettany-Saltikov (12). The research question developed in accordance with PEO was: Among marginalized individuals in Canada, how have social injustices experienced during the COVID-19 pandemic impacted their way of life? With regards to the research question; the population of the study refers to the marginalized groups residing in Canada, the ‘exposure’ involves the diverse forms of social injustices encountered by marginalized groups during the pandemic, and ‘outcome’ addresses the documented effects of these injustices on the lived experiences, health, and socioeconomic circumstances of affected communities.

#### 2.1.1 Defining the search keywords

Given the research question, getting the right keywords is essential in obtaining relevant publications from the database used. The search strategy included articles and editorial materials to get diverse opinions on social injustices experienced in Canada. Web of Science (WoS), Scopus, and PubMed databases were used in this study to ensure comprehensive, rigorous coverage of the interdisciplinary literature on pandemic-related social injustices. WoS database is considered as the world’s most trusted citation index for scientific and scholarly research and has over 21,000 peer-reviewed journals from various research areas Clarivate (13). Scopus, chosen for its broad multidisciplinary scope, provides a critical dataset that captures the intersectional nature of the study (14–16).

PubMed database was also included in the study for its unparalleled depth in public health research, ensuring robust coverage of health disparities, and clinical outcomes related to COVID-19 among marginalized populations (17–19). The multi-database approach aligns with the best systematic review practice by facilitating comprehensive literature identification, reducing the risk of missing relevant studies, and enhances the transparency of the review process (20). WoS, Scopus and PubMed databases provide a robust, triangulated evidence base that supports a nuanced synthesis of the social, structural, and health inequalities that was exacerbated by the coronavirus pandemic across Canada.

The keywords “social injustice”, “social justice challenges”, “social justice”, “health equity”, “prejudice”, “racism”, “social discrimination”, “health inequalities”, “social exclusion”, “structural racism”, “systemic racism”, “social justices issues”, “health inequities”, “economic disparities”, “marginalized”, “vulnerable”, “coronavirus”, “SARS-Cov-2”, and “COVID-19” were included in the search to cover a broad range of marginalized population. Synonyms of the keywords were also used to reach a broader database. The study applied various search syntax strategies to improve, expand and enhance the quality of keyword search. These include the Boolean operators such as “AND” and “OR,” phrases, truncation, and wildcard. Studies undertaken from 2020 to 2023 were considered in the study as it reflects the duration of the coronavirus pandemic. A total of 604 publications were gathered from the WoS database using the keyword strategy. One thousand eight hundred and fifty-nine (1859) documents and 1,495 publications were also retrieved from Scopus and PubMed databases for the study, respectively. In addition, five (5) articles from other open data sources. Titles, abstracts, and keywords were all utilized in this stage of the search query.

### 2.2 Screening

In the screening stage, studies were included if they were published between 2020 and 2023, focused on Canada as the study setting, examined social injustices or structural inequities experienced during the coronavirus pandemic, and centred on marginalized groups. For the purposes of the study, marginalized groups include indigenous people, racialized communities, 2SLGBTQ+ individuals, women, children, immigrants, refugees, older people, people with disabilities, and other equity deserving populations. Studies that were purely clinical without an explicit social justice framework were excluded. Studies that were not published in English, and studies without abstract were excluded in this study. Following the same screening criteria, 1859 records were initially retrieved from the Scopus database, and 1,495 records retrieved from PubMed database. Out of the 3,963 records that were merged from all the databases, 3,651 records duplicates were removed, and 57 papers were excluded for reasons like irrelevant title and language exclusion in the Identification stage.

In the Screening stage, 250 documents were eligible for the title and abstract screening. A total of 43 papers were excluded from the dataset as they were irrelevant to the research question. As a result, 207 papers qualified for full text review at this stage. Fifteen (15) papers were excluded from the study because the full text documents were unavailable after exhaustive attempts to retrieve them from institutional and library resources. After a carefully full text screening of the 192 documents; 12 papers were excluded from study for focusing on ineligible population, 31 papers were excluded due to examining exposures that were irrelevant to the research question, and 91 studies were also excluded because their outcomes did not align with the research question. The screening stage was done independently by authors to enhance objectivity and reduce selection bias. After the independent screening, authors compared decisions to resolve any discrepancy.

After the identification, screening and the eligibility assessment stages, 58 papers met the inclusion and exclusion criteria and were added to the final review of the study.

### 2.3 Eligibility

The full text of all the 250 papers was retrieved and assessed independently by authors against the inclusion and exclusion criteria established for this study. This section of the PRISMA framework provides criteria for inclusion and exclusion of publications considered in the study. For a paper to be included and considered for a full text review, it must be from the period 2020–2023 to cover the period of the COVID-19 pandemic. The publications considered are articles, editorial materials, and reviews. Empirical (quantitative, qualitative, and mixed) and conceptual papers related to social injustices pertaining to Canada were considered in the full text review.

**Figure 1:**
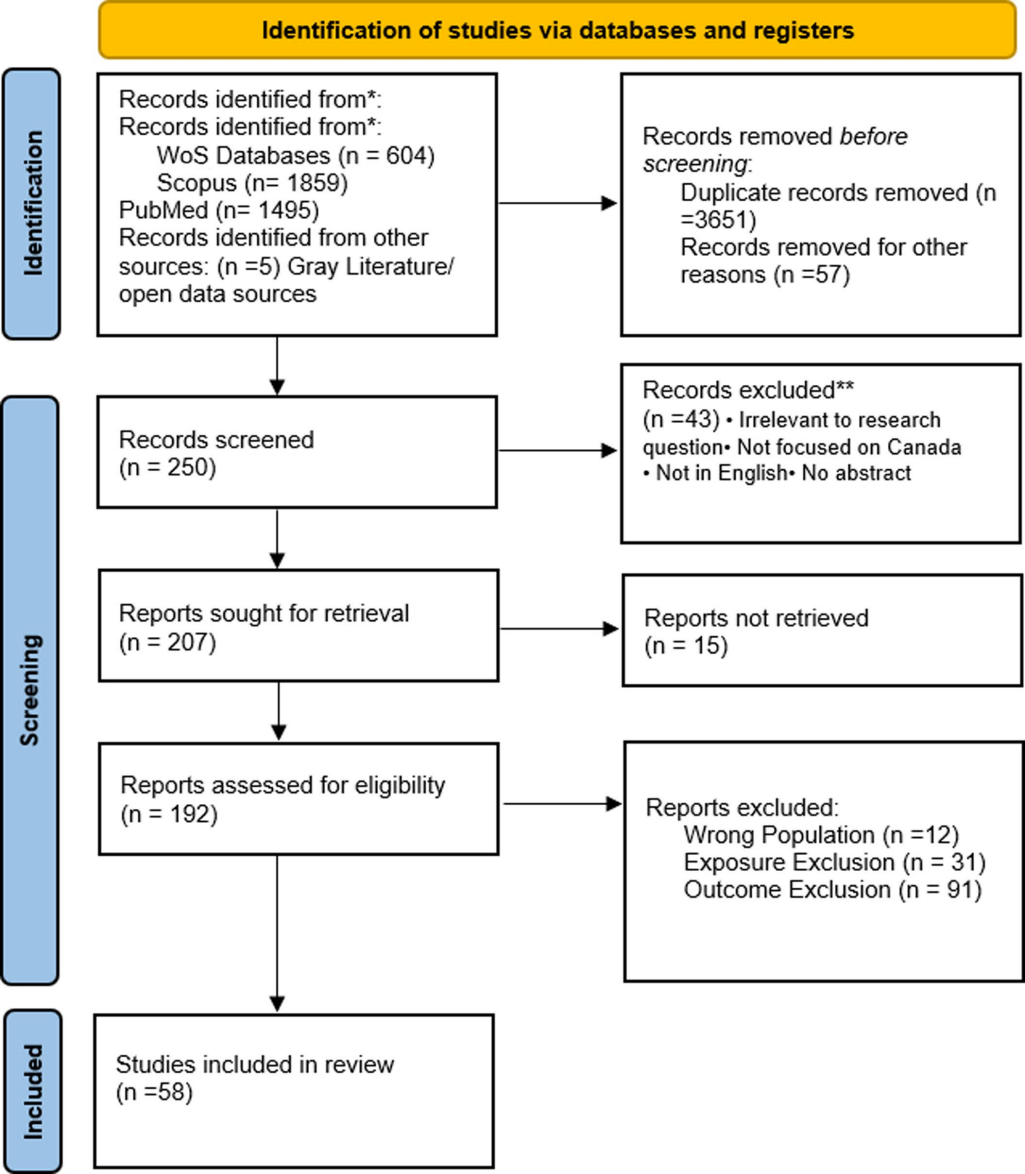
PRISMA 2020 flow diagram. Source: authors’ own compilation using PRISMA 2020 (11).

Published papers that included data from more than one country were excluded from the study. In addition, inaccessible full text publications from WoS were also excluded from the study. Papers published in any other language apart from English were also excluded from the study. Unsuitable papers or publications that did not cover issues surrounding social injustices were not considered in the study. Figure 1 is a PRISMA 2020 flow diagram that gives a pictorial view of the selection process, from initial identification to final inclusion.

### 2.4 Quality assessment

The quality of the methodology of the 58 selected papers was assessed independently by both authors using the Mixed Methods Appraisal Tool (MMAT) version 2018 (21). MMAT was selected because of its strength in allowing for the simultaneous appraisal of qualitative, quantitative, and mixed-method studies. This unique strength of the tool aligns with the diverse methodological approaches in the selected literature. All the selected 58 studies were evaluated against five core criteria specific to its methodological category and categorized as follows:

*Category 1***—**Qualitative

*Category 2***—**Quantitative randomized controlled

*Category 3***—**Quantitative non-randomized

*Category 4***—**Quantitative descriptive

*Category 5***—**Mixed Method

In each category, MMAT assesses appropriateness of the sampling strategy, measurement tools, and coherence between the research objectives and methodology. For the purposes of this review, papers were not excluded based on quality scores, rather, this appraisal provided a contextualized assessment of the strength of evidence underpinning the findings in this study, identifying areas of methodological rigor and limitation across literature. Both authors independently assessed each of the 58 selected studies on all the categories against the relevant MMAT criteria, and disagreements were reconciled through discussion. The inter-rater reliability between the authors was high (*k* = 0.84) with all discrepancies resolved through consensus discussion.

Out of the 58 included studies, editorial material (*n* = 1) was included to capture expert commentary and policy positions that contextualize empirical findings. MMAT criteria were applied to the editorial material with the understanding that they function as expert opinion rather than empirical evidence. The contribution from the editorial material to synthesis was weighted accordingly.

#### 2.4.1 Criterion-by-criterion results

Table 1 provides a summary of results for the criterion-by criterion assessment.

**Table 1:** Results from the criterion-by-criterion assessment.

Criterion	Number of papers that met the criterion	% Met
Qualitative studies (*n* = 32)		
Qualitative approach and research questions appropriate	32/32	100.0%
Data collection methods are adequate to address the research question	31/32	96.9%
Findings adequately derived from the data	30/32	93.8%
Interpretation of results sufficiently substantiated by data	29/32	90.6%
Coherence between data sources, collection, analysis, and interpretation	29/32	90.6%
Quantitative descriptive studies (*n* = 18)		
Sampling strategy relevant to address the research question	17/18	94.4%
Sample representative of the target population	14/18	77.8%
Measurements appropriate regarding exposure/intervention and outcomes	16/18	88.9%
Risk of nonresponse bias low	12/18	66.7%
Statistical analysis appropriate to answer the research question	16/18	88.9%
Mixed-methods studies (*n* = 8)		
Adequate rationale for using a mixed-methods design	8/8	100.0%
Different components of the study effectively integrated	7/8	87.5%
Outputs of the integration adequately interpreted	6/8	75.0%
Divergences and inconsistencies between quantitative and qualitative results adequately addressed	5/8	62.5%
Different components adhere to the quality criteria of each tradition	7/8	87.5%

Source: authors’ own assessment using the MMAT guidelines by Hong et al. (21).

### 2.5 Data extraction and thematic synthesis

Following the thematic synthesis approach developed by Thomas and Harden (22), the study applies a three-step approach: line-by-line coding of findings from the included studies, developing descriptive themes, and generating analytical themes guided by the objectives of the study. In the first step, findings from the 58 included studies were extracted verbatim and codes were assigned. In the second stage, codes generated from stage 1 that are related are grouped into descriptive themes through iterative discussion by the authors. In the third stage, the descriptive themes are carefully analyzed, and new conceptual constructs are developed into interrogative themes.

Thematic saturation was assessed iteratively at every step of the coding process. By approximately the 40th included study in the dataset, no substantively new descriptive code emerged; the remaining papers confirmed the existing themes and did not generate new conceptual categories. The informal but systematic code monitoring system supports the credibility of the analytical themes retrieved from the study.

## 3 Results

Results will be analyzed in three categories: Descriptive analysis and literature classification, and the conceptual framework model to explain social injustices during the pandemic in Canada.

### 3.1 Descriptive analysis

The descriptive analysis comprises the examination of year-wise publication, journal-base publication, document composition, and an analysis of subject area that focused on social injustices in Canada.

#### 3.1.1 Year-wise publication

**Figure 2:**
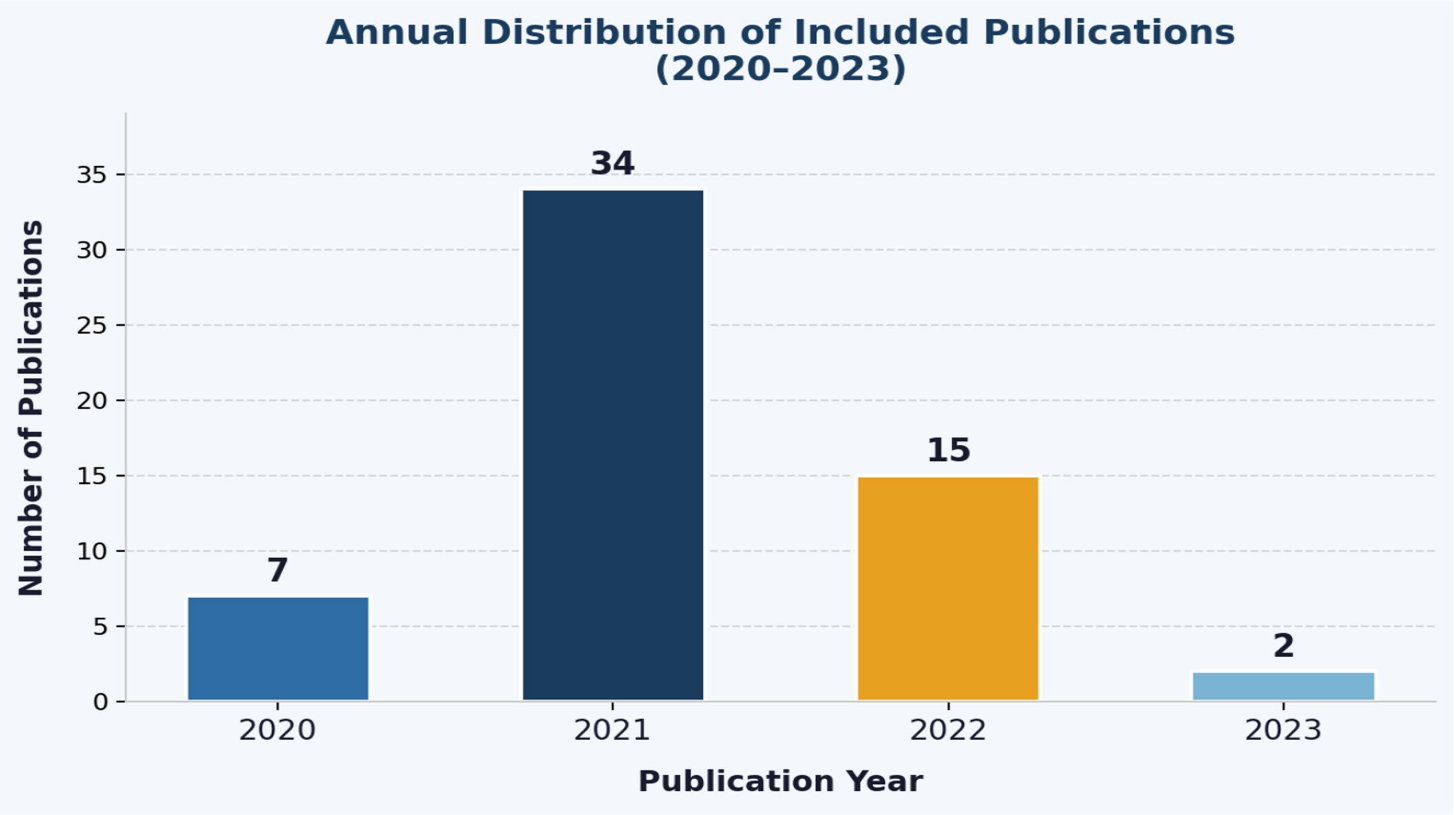
Annual publication distribution. Source: authors’ data from 58 selected publications.

The graph below shows the year-wise publication of social injustice research works published from 2020–2023. The publication distribution shows a pronounced surge in scholarly output during 2021 which accounts for approximately 58.6% of the dataset. The sharp increase in research activity may be attributed to the heightened public discourse on social injustices during the second and third waves of the pandemic. The pandemic exposed and intensified the many existing social inequalities in Canada and this prompted an increased scholarly attention on the issues of systemic discrimination, health inequities, economic inequalities and social exclusion. The decline in publication in 2022 and 2023 may be attributed to the transition of societies toward post-pandemic recovery. The decline may be partly attributable to publication lag, as studies that were initiated during later years may not have completed the peer-review and publication process at the time of data collection (see Figure 2).

#### 3.1.2 Document composition

**Figure 3:**
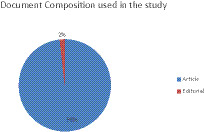
Document composition. Source: authors’ data from 58 selected publications.

Out of the 58 publications used in the study, about 98% of the documents were articles and 2% was editorial material (see Figure 3).

#### 3.1.3 Journal-base publication

Another important descriptive analysis is to examine the journals that contributed to the study. From the data analyzed in MS Excel, journals from different specializations covered various aspects of social injustices in Canada. This is a clear indication of the spread of social injustices experienced across varying fields during the pandemic. The journal with the highest contribution is the Canadian Journal of Public Health with 6 papers. The prominence of the journal in this study is consistent with the mandate to publish both empirical and policy-relevant studies on health disparities, making it a natural outlet for COVID-19 equity research. International Journal of Environmental Research and Public Health is the journal with the second highest research papers in the dataset. The table below displays the list of Journals with most contributions in the field of social injustice in Canada. The distribution across journals including Frontiers in Sociology, Studies in Social Justice, Qualitative Social Work, and Plan Canada highlight the interdisciplinary nature of pandemic injustice research. The journals focusing on sociology, social work, public policy, and community development suggest how the pandemic-induced injustices were examined from multiple disciplinary lenses and reflects the complex multifaceted nature of social inequality. Table 2 lists the top 10 contributing journals in the dataset.

**Table 2:** Top 10 contributing journals to social injustice in Canada.

Journal/source	Number of publications	%
Canadian Journal of Public Health	6	10.3%
International Journal of Environmental Research and Public Health	3	5.2%
Child Abuse & Neglect	2	3.4%
University of Toronto Medical Journal	2	3.4%
Frontiers in Sociology	2	3.4%
Plan Canada	2	3.4%
Qualitative social work	2	3.4%
Studies in social justice	2	3.4%
Canadian public policy	1	1.7%
Gender and society	1	1.7%

Source: authors’ data from 58 selected publications.

#### 3.1.4 Most cited publications

**Figure 4:**
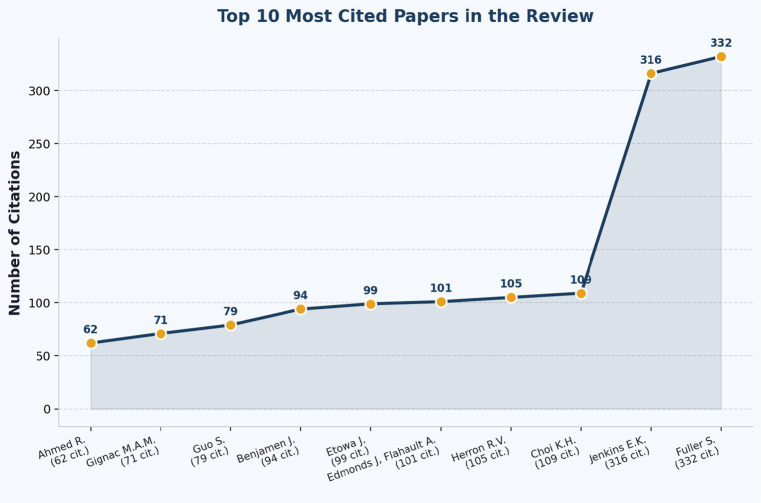
Top cited publications. Source: authors’ data from 58 selected publications.

The line graph below projects the most cited papers in this study. Based on the 58 selected publications, the highest cited paper is “Covid-19 and The Gender Gap in Employment Among Parents of Young Children in Canada” with 332 citations, was published by Fuller and Qian (8). This study serves as the cornerstone for intersectional pandemic research as it provides quantitative evidence that systemic inequalities which existed before the pandemic, were drastically exposed and intensified during the COVID crisis. The paper made a strong argument that women had to choose between their jobs and their children’s care during the pandemic. Jenkins et al. (9) is the second most cited paper with 316 citations and provides empirical evidence that supports the argument that COVID-19 did not affect everyone equally, but instead widened existing mental health inequities for Canada’s most marginalized populations. Choi et al. (7) is the third most cited paper and the study found that communities with larger shares of Black, refugee, and low-income residents had significantly higher rates of COVID-19 infection and mortality. The central thesis of Choi et al. (7) was the existence of meta-injustice; race-based data and other demographic information on COVID-19 patients were not collected consistently across Canadian provinces. The line graph below graphically displays the top ten (10) most cited papers being considered in this study. Table 3 provides the list of the top 10 cited papers in the study (see Figure 4).

**Table 3:** Top 10 most cited publications.

#	Authors	Title	Year	Citations
1	Fuller and Qian (8)	Covid-19 and The Gender Gap in Employment Among Parents of Young Children in Canada	2021	332
2	Jenkins et al. (9)	A portrait of the early and differential mental health impacts of the COVID-19 pandemic in Canada	2021	316
3	Choi et al. (7)	Studying the social determinants of COVID-19 in a data vacuum	2021	109
4	Herron et al. (65)	Conversations in times of isolation: Exploring rural-dwelling older adults’ experiences during COVID-19 in Manitoba	2021	105
5	Edmonds and Flahault (66)	Refugees in Canada during the First Wave of the COVID-19 Pandemic	2021	101
6	Etowa et al. (24)	Unpacking the health and social consequences of COVID-19 through a race, migration and gender lens	2021	99
7	Benjamen et al. (67)	Access to refugee and migrant mental health care services during the first six months of COVID-19: A Canadian survey	2021	94
8	Guo and Guo (68)	Combating anti-Asian racism and xenophobia in Canada: toward pandemic anti-racism education in post-COVID-19	2021	79
9	Gignac et al. (69)	Impacts of the COVID-19 pandemic on health, financial worries, and perceived organizational support among people with disabilities	2021	71
10	Ahmed et al. (70)	Racial equity in the fight against COVID-19: a qualitative study examining the importance of collecting race-based data in Canada	2021	62

Source: authors’ data from 58 selected publications.

### 3.2 Literature classification

The literature classification section entails detailed data analysis that answers the research question considered in this study. Based on the research question, the classification section will address the question under the following subheadings: Social Injustice, Marginal Communities, Theories/Framework used, and the impact of social injustices. A pandemic-induced social injustice intersectionality conceptual framework will be developed in this study to explain how overlapping social identities all must be considered in order to fully understand social injustice in Canada.

#### 3.2.1 Thematic synthesis

**Figure 5:**
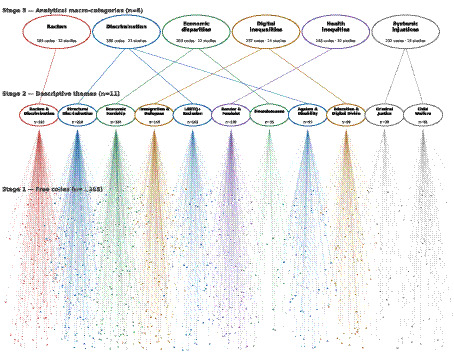
Thematic synthesis: social injustices during COVID-19 pandemic in Canada. Source: Authors’ own thematic synthesis compilation.

From the line-by-line coding of all the findings/results from the selected 58 studies, 1,385 descriptive codes were developed based on the central research question of this study. After the codes were generated, it was independently cross-checked by both authors to ensure analytical rigor, consistency, and credibility in the coding process. These descriptive codes formed the foundation of the second stage thematic synthesis where codes are arranged in clusters based on their conceptual similarity and underlying meaning to form themes. The average code per paper across categories was 8.43. While the study “Toward an Intersectional Approach to Health Justice” by Borras (23) received the highest number of codes (10), the study with the fewest number of codes assigned (3 codes) was “Strengthening the Collection and Use of Disaggregated Data to Understand and Monitor the Risk and Burden of COVID-19 Among Racialized Populations” authored by Etowa et al. (24). The second stage of the process involves constant comparison of codes to identify patterns, relationships, and points of convergence within the data. Eleven (11) descriptive themes were developed to represent the cluster groups formed as a result of conceptual similarity among the codes. Each theme constituted a set of related experiences, conditions, or systemic dynamics identified across the 58 studies. In the third stage, the descriptive themes were synthesized into 6 macro interrogative themes framed as an analytical proposition challenging assumptions about policy, identity, and power. The analytical/interrogative themes are the broad umbrellas of social injustices that will be further addressed through the development of a conceptual framework in this study. Figure 5 provides a pictorial view of the thematic synthesis process from line-by-line coding to analytical themes.

Table 4 gives a tabular breakdown of the coding process, illustrating the progression from Stage 1 (initial descriptive codes) through Stage 2 (development of descriptive themes) to Stage 3 (generation of analytical theme categories).

**Table 4:** Tabular representation of the theme development.

Analytical themes	Descriptive themes
Racism	Racism & anti-Asian/anti-Black/anti-Muslim racism
Discrimination	Structural discrimination & intersectionality; LGBTQ+ exclusion & cis-heteronormative policy; ageism, disability & digital exclusion of older adults
Economic disparity	Economic hardship & precarious labour; homelessness & end-of-life neglect
Digital inequalities	Education, digital divide & community pedagogy; immigration, refugees & transnational vulnerability (digital dimension)
Health inequalities	Gender inequality & feminist analysis; healthcare access barriers (BIPOC, immigrants)
Systemic injustices	Criminal justice, incarceration & abolitionist resistance; child welfare, youth marginalization & policy failure; immigration & refugee systemic vulnerability

Source: authors’ own thematic synthesis compilation.

**Figure 6:**
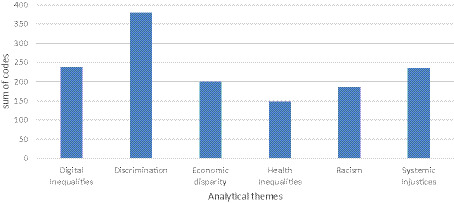
Graph of analytical themes and code composition. Source: authors’ own thematic synthesis compilation.

The graph of analytical themes and code composition shows that discrimination emerges as the prominent analytical theme with the highest number of codes (380), incorporating structural discrimination, intersectionality, LGBTQ+ exclusion, cis-heteronormative policy frameworks, as well as ageism and the digital exclusion of older adults. This finding suggests that discrimination is a central theme of injustice discussed by researchers and is one of the most pervasive and multilayered forms of social injustice in Canada during the COVID-19 pandemic. The high number of code concentration points to the systemic and embedded nature of discrimination across multiple social, institutional, and policy contexts. It further suggests the pandemic did not create new inequalities as much as it exposed, amplified and reproduced pre-existing structures of marginalization. The findings also shed light on the institutional failures of systems that disproportionately affect marginalized communities during the pandemic. The high clustering of codes within the systemic injustice theme displays how incarceration practices, youth marginalization, policy shortcomings, and structural vulnerabilities faced by marginalized communities are interconnected manifestations of broader systemic inequities. The interrogative theme of digital inequalities (codes = 237) highlights how unequal access to technology can shape how marginalized groups experience exclusion during the pandemic. With the draconian lockdown policies implemented by government, coupled with limited in-person services available during the pandemic, there was an intensified reliance on digital platforms for work, education, and access to essential services. As a result, limited digital access and literacy excluded many marginalized groups from fully participating as economic agents. The findings from the health inequalities’ theme highlighted the pre-existing structural inequalities within the Canadian healthcare system that were further exposed and intensified during the COVID-19 pandemic (see Figure 6).

#### 3.2.2 Social injustice

**Figure 7:**
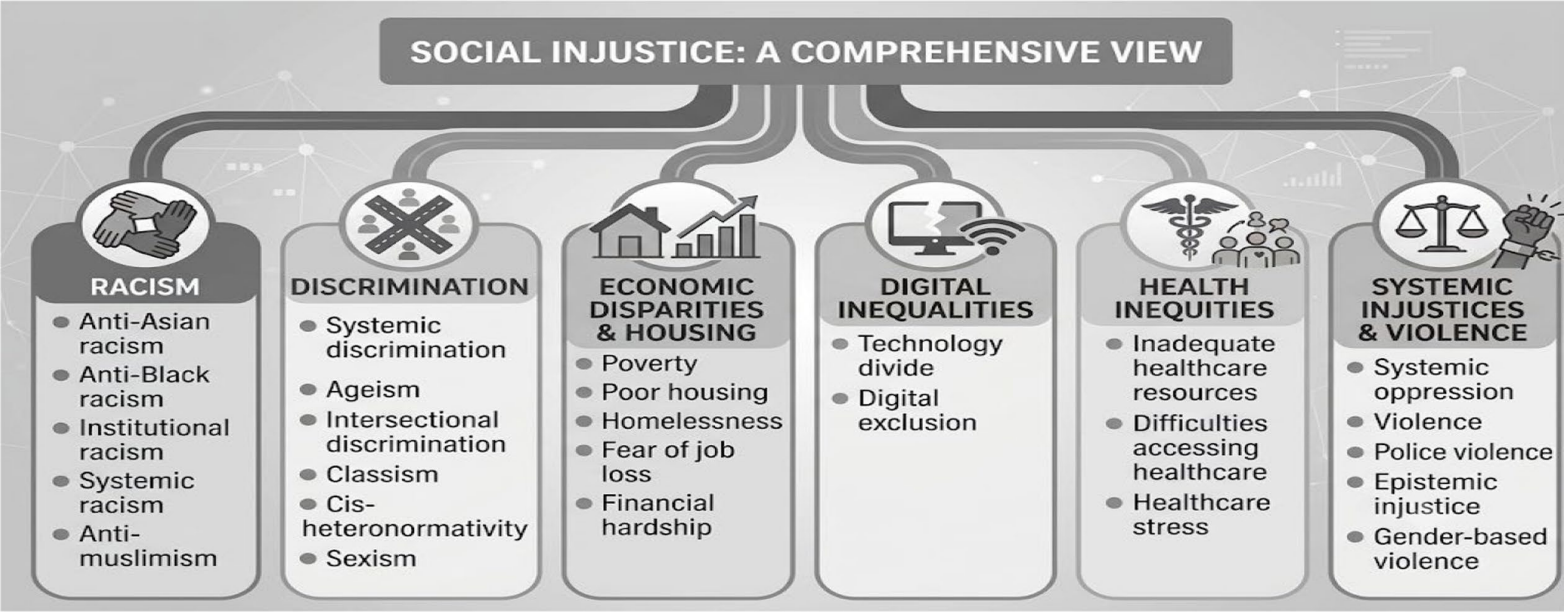
Social injustice diagram. Source: authors’ own compilation through the systematic review data.

In the book “Social Injustice and Public Health” by Levy and Sidel (25), Social injustice is explained from two perspectives. The first perspective considers the denial of human rights (economic, sociocultural, political or civic rights) of a specific group of people based on a fallacious perception of their inferiority by those with more influence as social injustice. The second definition refers to policies or actions that adversely affect the health conditions of a society. Based on data gathered from 58 publications on social injustice in Canada during the pandemic, a number of social justice challenges were analyzed. The social injustices discussed by researchers during the recent pandemics can broadly be put under 6 umbrellas: Racism, Discrimination, economic disparities, digital inequalities, Health inequities and Systemic injustice. The diagram below gives a breakdown of the social injustices covered in this study from 2020 to 2023 (see Figure 7).

#### 3.2.3 Marginalized communities

**Figure 8:**
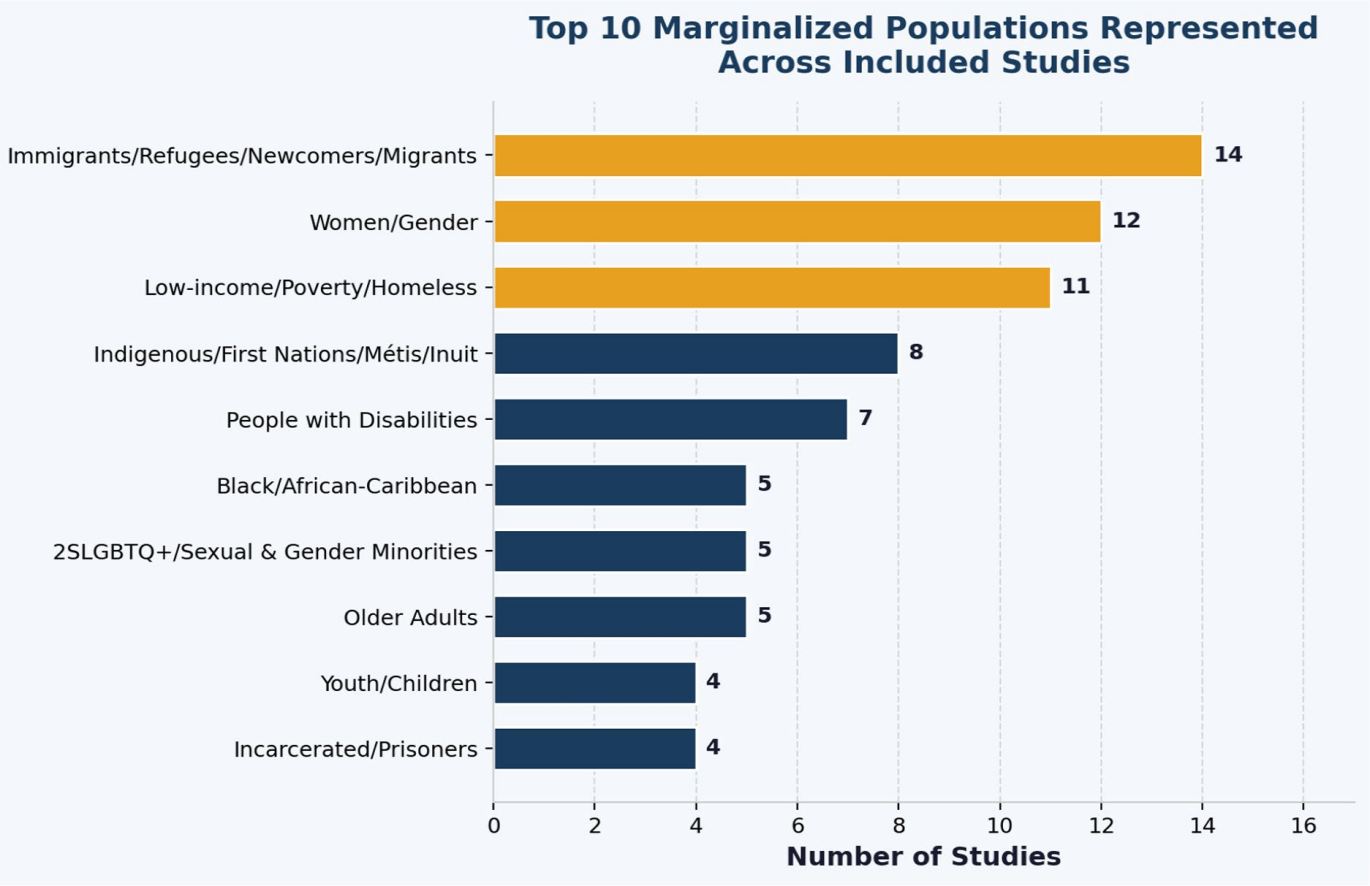
A clustered bar graph of the marginalized groups studied in the 58 publications. Source: authors’ data from 58 selected publications.

Social Injustice in Canada during the pandemic was experienced by different groups of people at varied degrees of intensity. The different marginalized groups reflect the various social identities an individual can have and how social injustices can affect identities. Sevelius et al. (64) explained marginalized communities as groups excluded from mainstream social, economic, educational, and/or cultural life. Marginalized communities are classified according to race, gender identity, sexual orientation, age, physical ability, language, and immigration status. Racialized groups were one of the groups profoundly impacted by the pandemic. Komeiha et al. (10) argued in their work that, racialized Canadians experienced a multifaceted impact of the pandemic, encompassing not only health outcomes but also severe social and economic consequences (see Figure 8).

The clustered bar graph (Frequency of marginalized groups discussed) gives a pictorial view of all the various marginalized groups discussed in this paper to have been impacted by various social injustices during the pandemic. The bar graph displays a graphical view of the marginalized groups discussed within the 58 selected publications of the study.

The intersectional nature of social identities of people makes them vulnerable to one or many social injustices discussed in this paper. From the horizontal bar graph, Immigrants, Refugees, Newcomers, and Migrants constitute the most frequently studied group, followed by women group and low-income group, respectively. It is important to note that a woman, who identifies as a Black person, an immigrant, has a prison record, a sex worker and is lesbian may experience different forms of discrimination at different levels, and different degrees based on how the society views them at a particular point. The intersecting nature of different identities from marginal groups creates unique and complex experiences of disadvantage that cannot be fully understood by examining each identity in isolation. Marginalized groups like the indigenous communities are visible in the bar chart, appearing in eight studies of the dataset. This suggests that policy makers and researchers recognized how disproportionately indigenous communities were impacted during the pandemic. This study specifically delineated the Black community within this demographic to elucidate the distinct impact of social injustice experienced by Black Canadians. The diverse multilayered marginal groups that experienced social injustices during the pandemic reflect the high level of risk exposure to social injustice a Canadian face while keeping in mind the intersectionality of different social identities an individual can have at a particular point. The frequencies presented in this chart cannot be interpreted as mutually exclusive categories, as several papers analyzed the interconnected experiences of multiple marginalized social identities.

#### 3.2.4 Theories/framework

**Figure 9:**
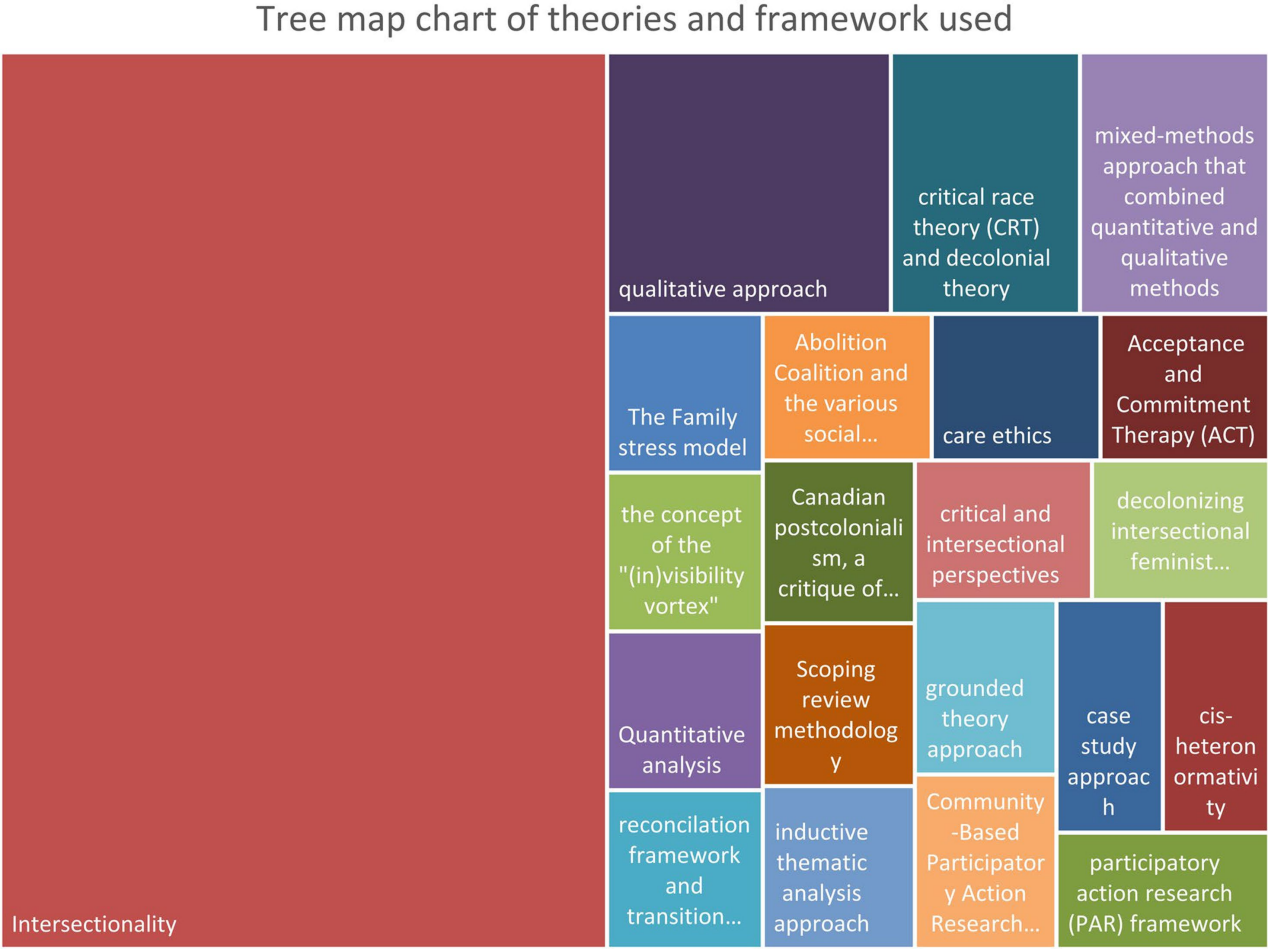
Tree map diagram of theories and framework used in the data. Source: authors’ data from 58 selected publications.

Numerous frameworks have been used to explain the phenomena of social injustices in Canada during the pandemic. About 47% of the studies applied the varied forms of intersectionality to understand how social injustice can impact an individual due to race, sexual orientation, gender, religion, language, immigration status, physical ability, etc. Crenshaw (26) explained the concept of intersectionality as various ways in which race and gender interact to shape the multiple dimensions of Black women’s employment experience. Since its inception in the 1990s, the intersectionality framework has been adopted and adapted to explain several complex overlapping issues. For example, in the paper “Identity, politics, and the pandemic: Why is COVID-19 a disaster for feminism(s)?,” the intersectionality framework was used to explain why gender alone is inadequate in addressing the risk and consequences of COVID-19. It explained how indigenous women and Black women were faced with increase in COVID infection and how delayed healthcare and limited quality care impacted indigenous women and Black women (27). Some studied utilized the critical race theory (CRT) and decolonial theory to examine institutional, structural, and systemic racism in the school system and its impact on marginalized communities (28). In total, there are about 21 different frameworks adopted by researchers for a wide array of social justice analyses faced by marginalized groups. The tree map chart below displays all the various theories/frameworks that were applied in the respective studies (see Figure 9).

#### 3.2.5 Impact of social injustices among marginalized communities

Marginalized communities faced a myriad of social injustices that worsened disparities in health, economic opportunity, and their overall wellbeing. A careful analysis of data reveals that marginalized groups including indigenous people, racialized communities, immigrants, LGBTQ2S+, etcetera, bear disproportionate burdens of inequalities and this section of the research breaks down the impacts on the disadvantaged groups.

##### 3.2.5.1 Child welfare involved families, and indigenous, Black, Latinx, Southeast Asian women

Child welfare families during the pandemic experienced heightened rates of child maltreatment and neglect. Children and youth in Canada also faced increased vulnerability to family violence as a result of the various social injustices they experienced. Indigenous, Black, Latinx and Southeast Asian women faced limited quality healthcare, experienced increase in COVID infection, maternal stress, isolation and death in some cases. The intersectionality of gender and ethnicity heightens their vulnerability and magnifies the adverse impact of social injustice. Some women were harassed for wearing hijabs and others lost their jobs as a result of facing diverse social injustices in Canada.

##### 3.2.5.2 Entrepreneurs from marginalized backgrounds

Entrepreneurs from marginalized communities like rural and northern women, immigrant women, and indigenous women encountered systemic barriers that hindered their effective participation in mainstream entrepreneurship. Though the Women Entrepreneurship Strategy program which targets more than $2bn CAD in investment towards women businesses has made tremendous progress, technology and digital divide, and structural inequalities were heightened during the pandemic. The limited support system for women entrepreneurs that considers their ethnic background led to revenue loss fostering cycles of economic disadvantages (29).

##### 3.2.5.3 Employment, education, health disparities, and criminal justice inequities

During the pandemic lockdown, the disparities in employment, education, healthcare and the criminal justice system widened. From the data analyzed, it mostly affected indigenous people, women, members of the LGBTQ2S+ community, Black people, newcomers and rural folks. A paper by Marks and Toye (30) revealed that the pandemic laid bare the systemic and institutional racism that exist in the social, economic and criminal justice systems in Canada. These social injustices foster economic marginalization, hinder opportunities for growth, and perpetuate cycles of poverty and disenfranchisement in Canadian communities.

##### 3.2.5.4 Assault, alienation, high rate of morbidity, and mental health impact

The LGBTQ2S+ community experienced verbal assaults, experienced a sense of fear and vulnerability, high rate of morbidity and death during the COVID pandemic as a result of several social injustices meted at them. Some felt alienated and experienced isolation as a result of rejection from some French speaking communities and other minority groups (31). The stigmatization and the unwelcoming nature of some Canadians towards the LGBTQ2S+ community and the restricted social gathering during the pandemic affected the mental health of many in the rainbow community leading to depression, loneliness and other psychological health challenges.

##### 3.2.5.5 Homelessness and vulnerability

During the pandemic, homeless people faced neglect in the public health protocols, and this heightened their vulnerabilities during the pandemic (32). Homeless individuals often experience stigmatization which furthers adverse health outcomes (33). Healthcare inequities faced by homeless individuals increased the risk of mortality and amplified mental health struggles.

In summary, the findings from this study revealed the pervasive impacts of social justice struggles on marginalized communities in Canada. The impacts varied from economic exclusion to criminal justice inequities, health disparities, and systemic barriers fuel cycles of inequality. The road map addressing the diverse forms of social injustices discussed in this paper requires a comprehensive intervention that considers the intersectionality of multilayered social identities. To understand the impacts of social injustices, one must appreciate the complexities of intersectionality which allow an individual to face several injustices as a result of the social identity they carry at a point in time.

### 3.3 The conceptual framework of the Pandemic-Induced Social Injustice Intersectionality Model (PISIIM)

The findings from this systematic literature review illuminate the various social injustices that Canada experienced during the unprecedented global pandemic and its impact on women, children, the disabled, persons of colour, and many minority groups. Increase in family violence, health disparities, mental health deterioration, and assaults were some of the impacts of social injustice faced by the minority groups. These outcomes of social injustices did not arise in isolation, rather emerged as a result of the complex interaction of pre-existing structural vulnerabilities, pandemic-induced institutional disruptions, and overlapping social identities.

A conceptual framework is suggested by the study to theorize the dynamics in a coherent and analytically rigorous manner. This study proposes the Pandemic-Induced Social Injustice Intersectionality Model (PISIIM) to explain this phenomenon. By definition, a conceptual framework is explained as the roadmap in comprehending how the systems and/or variables interact to provide an answer to a social phenomenon (34). The PISIIM integrates multiple theoretical foundations to construct a comprehensive explanatory architecture that is robust and analytically coherent.

A novel contribution PISIIM adds to the social justice literature is that, the model does not merely position COVID-19 as a backdrop but as an active structural shock that intersects with, amplifies, and reshapes pre-existing inequalities. In addition, PISIIM provides a structure for stakeholders to anticipate and mitigate the insurgence of social injustices during future pandemics.

#### 3.3.1 Foundational theoretical Lens

The anchor theory for this framework is intersectionality, as proposed by Crenshaw (35). The theory explains how a person with multiple social identities intersect to create a unique experience of privilege and oppression. In the article “Mapping the Margins,” Crenshaw used the concept of intersectionality to expound on how the overlapping nature of social identities contribute to violence against women of colour. Bailey et al. (36) expanded the concept of intersectionality by analyzing it both as a form of critical praxis and analytical framework within broader power matrices of structural, disciplinary, hegemonic, and interpersonal domains. This elaboration strengthens PISIIM’s multi-level explanatory capacity in understanding the rise of injustices during the pandemic.

In applying intersectionality in this study, the proposed conceptual framework seeks to explain how marginalized individuals experienced social injustices during the COVID pandemic as a result of their intersecting identities.

In order to understand the structural dimensions of intersectionality, PISIIM adopts the Critical Race Theory (CRT). As championed by scholars like Derrick Bell, CRT argues that racism is systematic and not just episodic as it is embedded in institutions, policies and social norms (37). CRT provides a profound understanding of the increased disparities in healthcare access, employment security, and housing, during the COVID pandemic as rooted in pre-existing structural inequalities.

The Ecological Systems theory as explained by Bronfenbrenner (38), is instrumental in explaining how disruptions spread across the multi-leveled system (micro, meso, exo and macro systems) (39). Bronfenbrenner’s model provides insight into how individuals from the marginalized groups are influenced bidirectionally by the immediate environment (family, peers, neighborhood) (38). The mesosystem explains how the elements of the microsystem interact with each other. The Ecological Systems theory provides a theoretical foundation for the interaction at the workplace, media, public services (exosystem levels) with individuals in the marginalized groups bidirectionally in perpetuating social injustices during the COVID pandemic.

Galtung (1) developed a structural violence framework to capture the various forms of harm that are diffuse, systemic and often invisible as it is embedded and coded in social structures rather than enacted by individual perpetrators. Farmer et al. (2) advanced Galtung’s framework through the linking of structural violence explicitly to health inequities and argued that dissimilar health outcomes among disadvantaged groups are a product of socially arranged and historically constituted forces. PISIIM adopts this concept as it considers health disparities as an outcome variable deeply rooted in structural conditions rather than individual behavior.

Another important theoretical concept adopted in PISIIM is the Capabilities Approach as suggested by Sen (71) and Nussbaum (40). The Capabilities Approach posits that social justice is achieved when individuals have real opportunities (capabilities) to live meaningful and dignified lives. This approach provides a normative anchor for measuring social injustices as it represents a deprivation of capabilities; the freedom to be healthy, to participate in public life, to live without fear of violence and to access education. The Capabilities Approach is of particular importance to PISIIM because it shifts the evaluative focus from formal equality (equal treatment under law) to substantive equity (equal real-world opportunities).

#### 3.3.2 Variables

The variables in this model represent key constructs that can vary across individuals, marginalized groups, and social systems, and can influence, mediate or moderate the processes through which injustices are intensified during pandemics. The model contains structural antecedent variables (intersectional identity position, pandemic structural shocks), mediating barrier variables, moderator variables, dependent or outcome of inequality variables (see Table 5).

**Table 5:** PISIIM variable architecture summary.

Variable type	Name	Key constructs	Theoretical anchor
Structural antecedent	Intersectional identity position	Race, gender, age, indigeneity, disability, immigrant status, sexuality	Intersectionality theory (26, 62)
Structural antecedent	Pandemic structural shock	COVID-19 spread, lockdown policies, economic shutdown, social isolation	Ecological systems/chronosystem (38)
Mediating barrier	Institutional system disruption	Healthcare, justice, employment, family, community, information environments	Structural violence (1, 2)
Moderating variable	Structural intensifiers	Socio-economic status, digital access, institutional trust, pre-existing structural racism, misinformation exposure	CRT (37); capabilities approach (71)
Outcome of inequality	Pandemic-induced social injustices	Health disparities, family violence, mental health deterioration, housing insecurity, discrimination, barriers to justice	Capabilities approach (40); structural violence

Source: authors’ own derivation based on theories adopted.

##### 3.3.2.1 Independent variables

The first structural antecedent variable in this model is the intersectional identity position. Drawing from the intersectionality theory, this variable refers to the structural location of marginalized individuals within overlapping social identities such as race, gender, age, indigeneity, disability status, immigrant status, and sexuality.

The second structural antecedent variable represents the structural shocks that occur when a pandemic occurs. It is the external crisis event as a result of the COVID-19 virus spread, lockdown policies during the pandemic, economic shutdown, social isolation and fear.

##### 3.3.2.2 Mediating barriers

The mediating (system disrupting) variable captures the institutional disturbance triggered by the COVID-19 pandemic across key social systems, including healthcare, justice, employment, family, community networks, and information environments. These institutional systems disruptions act as mediating mechanisms that transmit the effect of the pandemic shock and intersectional vulnerabilities into heightened social injustices across marginalized groups.

##### 3.3.2.3 Moderating variables (intensifiers)

Moderating variables are structural condition variables that influence the strength or direction of the impact of social injustices among marginalized groups. Intensifiers like socio-economic status, digital access, trust in institutions, and pre-existing structural racism are conditions that help in explaining why some marginalized populations experience greater social injustice than others.

##### 3.3.2.4 Dependent variables (outcomes of inequality)

The dependent variable is the pandemic-induced social injustices representing the unequal social consequences experienced by marginalized individuals during the COVID-19 pandemic. This is the primary outcome of the model and is reflected in manifestations such as racism and ethnic discrimination, health disparities, family violence, mental health deterioration, housing insecurity, barriers to justice, unemployment, and social stigmatization.

#### 3.3.3 How PISIIM works

PISIIM is a dynamic multi-level pathway explanatory framework which starts with two structural antecedent variables interacting at the intersection of identity and crisis. The intersectional identity position which is the first structural antecedent variable represents the structural location of an individual. The social identities of the individual overlaps and interacts to produce distinct vulnerabilities. For example, an African-Canadian child, disabled and belongs to the LGBTQ+ is likely to face different forms of oppression that comes with each social identity and an overlap of these identities can also create different forms of discrimination.

As nations grappled with the spread of coronavirus, draconian lockdown measures were implemented leading to increased social isolation, and this slowed down economic growth. The interaction between the pandemic structural shock (herein referred to as shock) and the intersectional identity position is unidirectional as the ‘shock’ flows through mediating variables for marginalized groups to experience injustices unequally. Borrowing from the Ecological systems theory as suggested by Bronfenbrenner (38), the mechanism can be explained through a defined sequence of steps that permeate across multiple, nested levels of the social environment simultaneously. When the COVID-19 pandemic struck Canada, it disrupted institutions like the family, healthcare, employment, justice, and community. For family systems as a mediating barrier variable, the shock through the lockdown measures confined family members to shared domestic spaces for prolonged periods, dramatically increasing household tensions and conflict (41). As businesses were shut and global supply chain was brought to a halt during the pandemic, the financial stability of families was undermined and this contributed to chronic stress that escalated to violence at home (42). Due to the strict lockdown measures, families found it difficult to access support programs and social services. This restricted access left victims without the intervention networks they previously depended on (43). As alcohol abuse significantly increased during social isolation and lockdown measures, the closure of schools also increased the psychological strain on parents. This created an environment for increased domestic violence (44).

Once the pandemic structural shock hit the healthcare systems of Canada, resources were prioritized towards COVID-19 management, resulting in the suspension of elective procedures and non-emergency care that marginalized groups disproportionately rely on (45). Black people, Indigenous, and racialized groups experienced disproportionately high rates of pandemic diagnosis, hospitalization and deaths as a result of pre-existing structural barriers that exist in accessing healthcare in Canada (46). Lack of culturally competent care, language barriers, and financial cost of taking time off work to seek hospital care are some of the structural barriers stemmed from the colonial design of healthcare. Misinformation and disinformation, coupled with the pre-existing vaccine trust among the Black communities delayed care-seeking and reduced vaccine uptake at critical moments in the pandemic (46, 47).

The pandemic shock impacted the employment system by triggering massive layoffs in the hospitality, retail, food service, care work, and agricultural labour which is dominated by racialized, female, and immigrant workers in Canada (48). The overlapping social identities make the level of financial loss impact vary across social identities. Essential workers, mostly people from racialized backgrounds were not offered the luxury of working remotely and therefore were exposed to the coronavirus as they worked to fend for themselves with little to no protective gear (44).

The pandemic structural shock also impacted marginal populations through the Justice systems in Canada. As courts were closed, women and other vulnerable populations trapped in violent homes lost their immediate recourse (49). Protection orders and emergency restraining orders were often delayed thereby deepening the woes of the vulnerable population (42).

As the pandemic heightened, lockdown restrictions on community gathering, social events, and religious centres dismantled communal support systems that marginalized people heavily relied on to battle loneliness, anxiety and depression (50). Racialized groups struggled to pay rent and mortgage as housing insecurity intensified during the pandemic (51).

The simultaneous activation of system failures through the pandemic institutional shock is what Galtung (1) described as structural violence perpetuated through systems. To understand the severity and direction of the impact of the pandemic shock on marginalized groups, it is important to understand the role of moderating variables (intensifiers) through the lens of Capabilities Approach theory. The Capabilities Approach provides an evaluative lens that measures what people could no longer do or be during the pandemic. It is important to note that the pandemic did not simply reduce income and resources, it dismantled conditions that made certain capabilities possible. Marginalized groups did not all enter the pandemic crisis on the same footing; their capabilities to do and be were already in vulnerable positions and the intensifiers ensured that as systems failed, the least equipped group felt the impact, the hardest. For example, the pandemic shock forced many institutions online (telehealth, remote learning, remote work, and government relief programs primarily shifted online) (52). During this time digital connectivity shifted from being a supplementary feature of modern lifestyle to one of survival and a prerequisite of basic participation in the Canadian society. Low-income marginalized households who could not afford the cost of internet, did not have adequate devices, and are digitally illiterate were unable to book a doctor’s appointment, excluded from the remote job market and could not actively participate in online education. The disadvantages faced by the marginalized population did not start with the pandemic crisis, rather it was intensified by the moderating variable (digital accessibility). Institutional trust is a critical moderating variable in intensifying the pandemic shock across racialized communities. For indigenous people and the Black community, histories of systemic exclusion in governance, healthcare and social services already existed before the pandemic (48). From the Capabilities Approach lens, institutional trust (a pre-existing intensifier) placed certain capabilities out of reach to Black people and indigenous groups even when the formal structures to support them existed. Ultimately, when the pandemic shock destabilized healthcare, governance and social services in Canada, institutional distrust compounded the crisis and removed capabilities that had never been fully accessible to these marginalized groups from the beginning.

The interacting effects of the two structural antecedent variables which is transmitted through system disruption and magnified by intensifiers ultimately manifests in the pandemic-induced social injustices outcomes. Health disparities, mental health deterioration, family violence, housing insecurity, unemployment, discrimination are some of the many outcomes experienced during the pandemic era in Canada (48). The dependent variables (outcome) are not isolated misfortunes but are structured predictable results of a complex structure with pre-existing vulnerabilities amplified its impact on marginalized groups through moderating variables interacting with a shock of the pandemic.

A unique feature of PISIIM is its incorporation of a bidirectional feedback loop that captures how the outcomes (dependent variables) circle back to deepen the vulnerabilities of the disadvantaged groups at the core of the model. Social injustices are not just outcomes; they act as inputs to further worsen the state of a marginalized population. In other words, injustice outcomes experienced and intensified during the pandemic crisis do not simply end when the pandemic ends; they become embedded structural disadvantages the vulnerable people carry forward. An example to illustrate the feedback loop of PISIIM is when a racialized person experiencing housing insecurity (outcome) during the pandemic has no option but to live in an overcrowded condition, which increases his rate of exposure. A higher exposure to coronavirus has the potential of leading to job loss and as these disadvantages accumulate, they reinforce pre-existing inequalities making it harder to recover post-pandemic. Consequently, the housing insecurity experienced by the racialized individual has moved from being a temporary outcome into a persistent structural barrier that shapes his wellbeing and future capabilities.

The pandemic disrupted the social systems and as a result amplified social injustices like racism, discrimination, stigmatization, classism, health inequalities, homelessness, etc.

While the PISIIM framework effectively identifies the simultaneous disruption of family, healthcare, employment and community systems, a comparative analysis demonstrates that institutional mechanisms functioned with different levels of intensity and produced different inequality outcomes across social groups and provinces. For instance, the shift to digital service delivery acted as an intensifier as the lockdown measures were enforced, disproportionately excluded low-income, racialized and Indigenous populations in rural and northern provinces like Manitoba and Saskatchewan, where broadband infrastructure is underdeveloped (51, 52). In contrast, urban areas in Ontario and British Columbia, despite experiencing similar income disparities, benefited from marginally better community-based digital programs that partially reduced the exclusionary effects (52).

**Figure 10:**
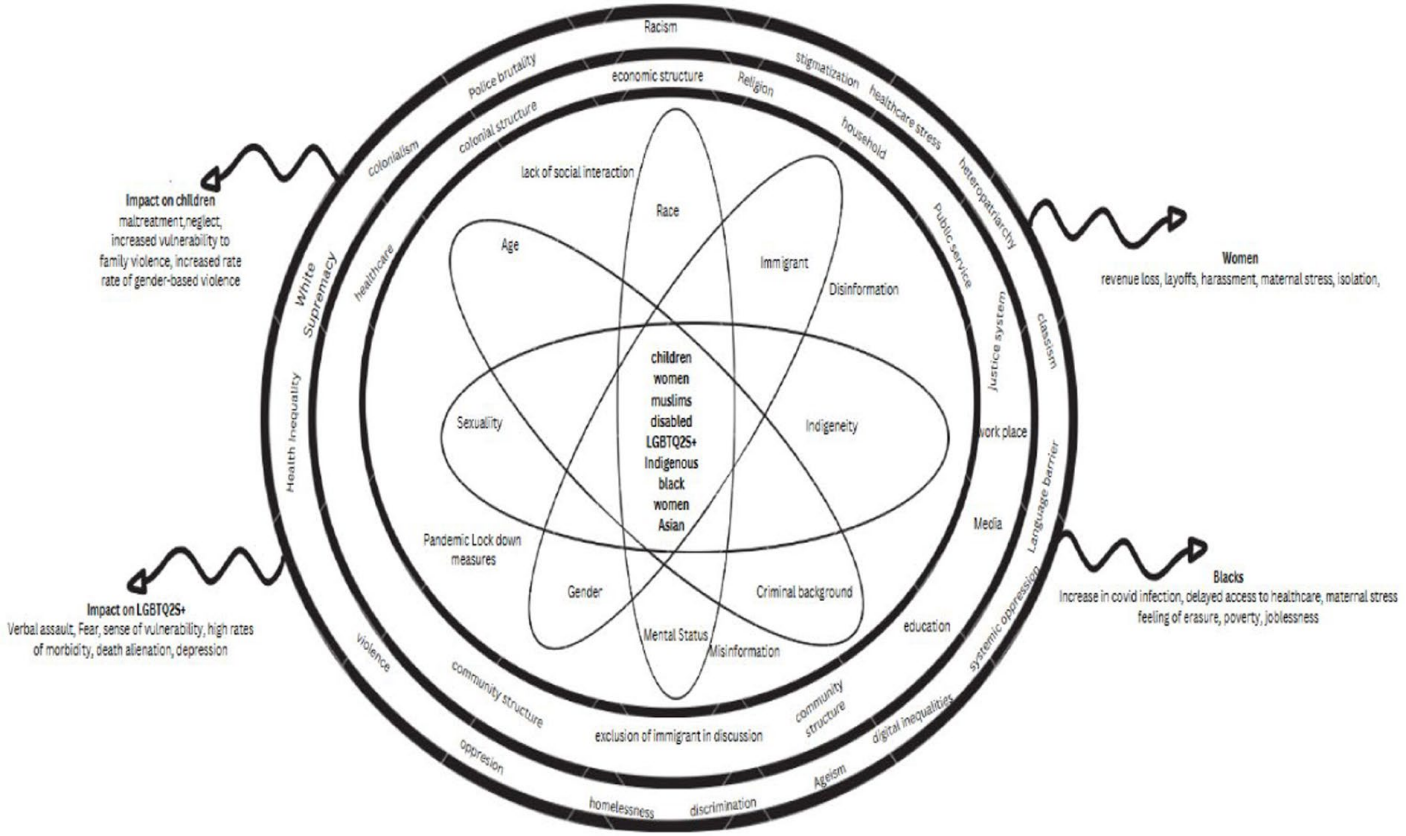
PISIIM framework. Source: author’s own design adopted from JASS intersectionality diagram (61).

Furthermore, comparing the employment and healthcare systems reveals a disjuncture between institutional impacts: the healthcare disruption produced direct, life-threatening health disparities through delayed care, while disruption in employment produced economic precarity that indirectly compounded housing and food insecurity. The former impacted the physical capability of an individual while the latter impacted the capacity to have a dignified livelihood. This comparison confirms that the COVID-19 pandemic did not produce a single uniform form of social injustice, but rather a range of different harms that are shaped by institutional systems and geographical locations (see Figure 10).

The diagram above provides a pictorial view of the conceptual framework. The framework is organized in three concentric layers that move from the centre outward. At the centre of the “virus looking” intersectionality model are the marginalized individuals that are intersected by race, age, sexuality, gender, criminal background, immigrant status, and indigeneity. The overlapping social identities create unique experiences of vulnerability and oppression.

The fluid space surrounding overlapping identity circles is the moderating variables (structural intensifiers) that determines the severity and direction of the pandemic’s impact on individuals.

The first outer circle after the fluid space represents the institutional systems disruptions triggered the COVID-19 pandemic. The disruptions in the healthcare, justice, family and employment systems acted as mediating barriers that transmitted the pandemic shock to marginalized communities. For instance, as the healthcare systems were overwhelmed during the pandemic, with resources diverted to COVID-19 management, this resulted in the suspension of elective procedure and non-emergency care that people for the marginalized community disproportionately relied on.

**Figure 11:**
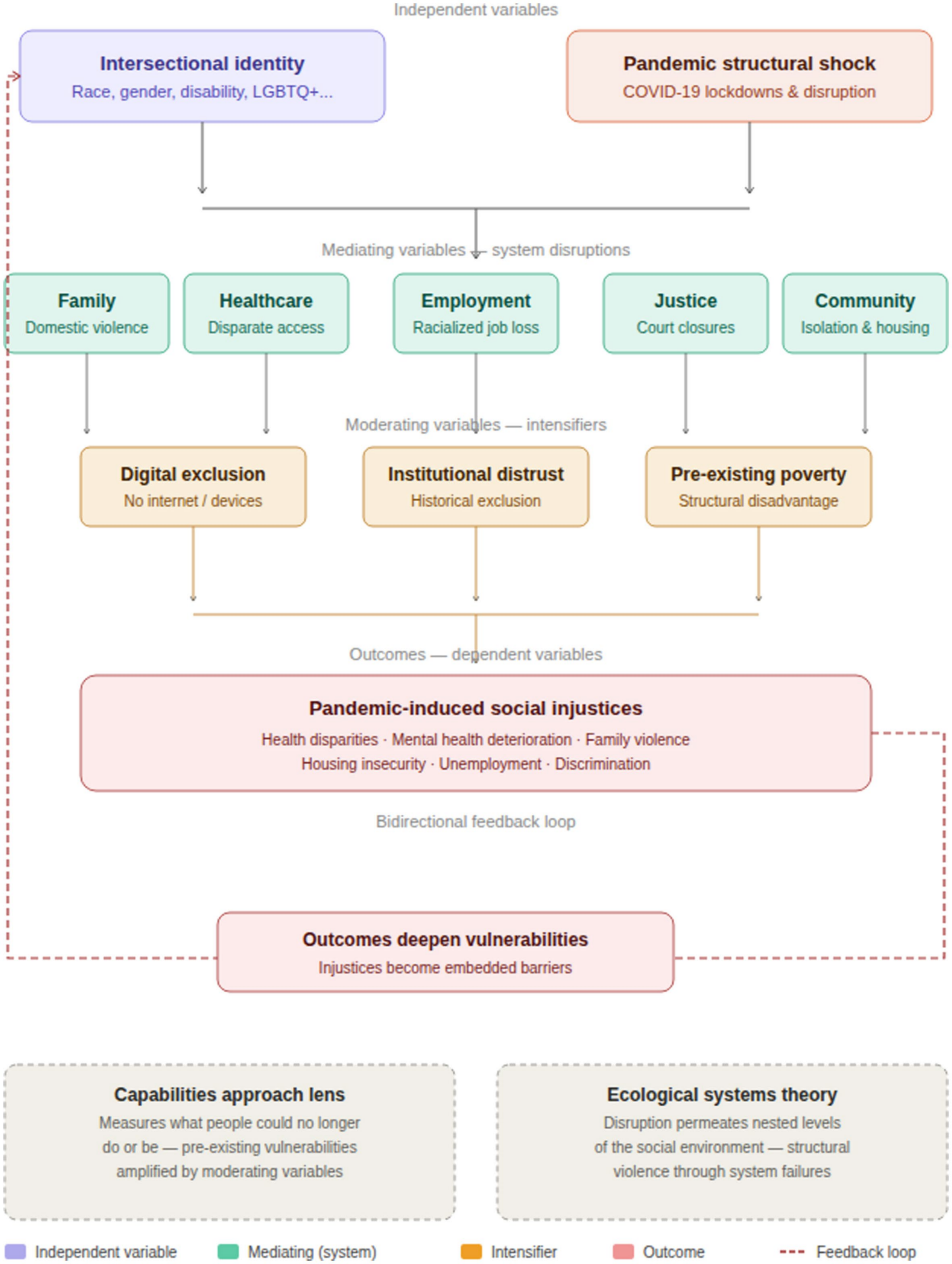
Mechanism of PISIIM framework. Source: authors’ own design.

The outermost circle represents the pandemic induced social injustice outcome which are the visible manifestations of how systemic failures filtered through moderating variables and landing on individuals with intersecting social identities. The wavy arrows reflect the nonlinear and uneven impact of the pandemic on the society (see Figure 11).

#### 3.3.4 Tensions and contradictions across literature

While thematic synthesis revealed clear patterns of social injustices across literature, a critical look of the studies also exposes important tensions and contradictions. There exist temporal variations throughout the 58 selected studies. Early pandemic research from 2020 to 2021 emphasized immediate health impacts and discrimination (7, 9), while later publications from 2022 to 2023 increasingly documented economic precarity, digital exclusion, and long-term mental health consequences (53, 54). A temporal shift in this manner suggests that the nature and primary mechanisms of social injustice literature evolved as the pandemic progressed. The temporal tension stated above reveals that pandemic-induced inequities are not static but dynamic, changing as institutional responses shift, resources are diverted, and cumulative disadvantages compound. The suggested PISIIM addresses this tension by incorporating a feedback loop that captures how social injustice outcomes become embedded as new structural barriers for future crises.

A second tension emerged in literature between quantitative and qualitative studies on the explanations of inequity. While quantitative studies in the dataset (*n* = 18) emphasize individual-level factors like vaccine hesitancy, health literacy, and income as inequity drivers, qualitative papers (*n* = 32) foregrounded structural determinants like systemic racism, institutional barriers, and colonial legacies as inequity drivers. The methodological tension reveals a deeper theoretical disagreement about the point of causation. PISIIM reconciles this by modeling structural conditions as antecedents, institutional mechanisms as mediating barriers, and individual capabilities as moderators, thereby integrating both perspectives.

Also, geographic tensions exist as about 70% of the included studies focused on Ontario and Quebec, with limited research from the Prairies, Atlantic Canada, and the territories. These tensions do not undermine the validity of the studies reviewed in this study, but reveals a complex, multifaceted and context-specific nature of the pandemic-induced inequities.

#### 3.3.5 Colonial structures and indigenous experiences

Unlike other marginalized groups whose vulnerabilities are shaped by policies on immigration and labour market stratification, the experiences of Indigenous people are embedded within colonial settlement systems characterized in the Indian Act, residential schools, and continuous violations of Indigenous sovereignty (55–57). These established colonial structures produce pre-existing inequities like housing overcrowding, food insecurity, and underfunded healthcare. As a result, these inequities were intensified during the pandemic (58).

Among the indigenous communities, the institutional disruptions identified in PISIIM operated differently. Disruptions in the healthcare systems were compounded by medical racism, limited access to culturally safe care, and distrust that is deeply rooted in colonial harms (59). The inexistence of disaggregated indigenous identity data also created an epistemic injustice that rendered indigenous experiences invisible in early pandemic response (7).

In spite of the many barriers faced by the indigenous groups in Canada, the community demonstrated strong resistance through community-led lockdowns, indigenous health authority mobilization, and vaccine distribution strategies (60). These indigenous strategies and responses underscore the need for indigenous sovereignty and self-determination as a pathway to social justice. Future research on PISIIM can focus on incorporating colonial structures as a mediating barrier and focus on indigenous-led governance during pandemic crises.

#### 3.3.6 Limitations and future directions

The following are the limitations and future directions for the PISIIM framework:

- The absence of a political economy lens: the PISIIM framework acknowledges system disruptions as a mediating mechanism through which the pandemic shock is transmitted. However, the framework does not account for the political and economic decision-making processes that shape how institutions respond or fail to respond to the needs of vulnerable individuals. A future study on PISIIM can look at how the integration of a political economy lens will enhance our understanding of the model.
- The treatment of Canada as a homogenous unit: the model treated Canada as a single unit for purposes of simplicity, but Canada is not a uniform landscape. It has provinces and territories with varying approaches to the pandemic, and different health and institutional systems. A future study can employ a heterogenous approach to Canada with emphasis on the uniqueness of the institutional systems across the different provinces.
- The absence of a theory that explains institutional response and governance failure: PISIIM does not account for theories of governance dynamics that examine how policies are constructed and which voices matter in policy deliberations.
- The model derived from systematic review is only theoretical and has not been empirically tested. Future studies can quantify the variables and develop appropriate empirical models to test the hypothesis.
- The model is silent on community resistance and resilience during the pandemic.
- Although the study employed three major multidisciplinary and health-focused databases (WoS, Scopus and PubMed) to ensure a robust and comprehensive systematic review of literature, the study acknowledges as a limitation that other relevant social justice databases exist and were not included in the search strategy. As a result, some pertinent studies indexed exclusively in specialized databases may not have been captured in the study.
- While the study provided a brief discussion on indigenous experiences during the pandemic, it does not provide the depth of analysis warranted by the distinct colonial-settlement systems that still shape indigenous vulnerabilities.

## Data Availability

The raw data supporting the conclusions of this article will be made available by the authors, without undue reservation.
